# Prevention and Control of African Swine Fever in the Smallholder Pig Value Chain in Northern Uganda: Thematic Analysis of Stakeholders' Perceptions

**DOI:** 10.3389/fvets.2021.707819

**Published:** 2022-01-13

**Authors:** T. Aliro, E. Chenais, W. Odongo, D. M. Okello, C. Masembe, K. Ståhl

**Affiliations:** ^1^Faculty of Agriculture and Environment, Gulu University, Gulu, Uganda; ^2^Department of Disease Control and Epidemiology, National Veterinary Institute, Uppsala, Sweden; ^3^Department of Zoology, Entomology and Fisheries Science, College of Natural Sciences, Makerere University, Kampala, Uganda

**Keywords:** African swine fever, disease control, focus group discussion, pork, prevention, northern Uganda

## Abstract

African swine fever (ASF) is endemic in Uganda and considered a major constraint to pig production. In the absence of a vaccine, biosecurity is key for ASF prevention and control. To improve prevention and control on farm and community level there is need for more knowledge on current application of biosecurity practises, and better understanding of how pig value chain actors perceive prevention and control. To achieve this, a qualitative interview study involving focus group discussions (FGD) was conducted with actors from the smallholder pig value chain in northern Uganda. Six villages were purposively selected based on previous outbreaks of ASF, preliminary perceived willingness to control ASF, and the representation of several different value chain actors in the village. Results indicated that biosecurity practises such as basic hygiene routines including safe carcass handling, minimising direct and indirect contacts between pigs or between pigs and people, trade restrictions and sharing of disease information were implemented in some of the villages. Thematic analysis based on grounded theory revealed six categories of data relating to ASF prevention and control. Together these categories form a logical framework including both enablers and hindrances for ASF prevention and control. In summary participants mostly had *positive perceptions of ASF biosecurity, describing measures as effective*. Participants further possessed *knowledge* of ASF and its transmission, some of which was in line with known scientific knowledge and some not. Nevertheless, participants were hindered from preventing and controlling ASF due to *biosecurity costs* and a need to *prioritise family livelihood* over disease transmission risks, incompatibility of current biosecurity practises with *local culture, traditions and social contexts* and finally lack of *access to veterinarians or, occasionally, low-quality veterinary services*. The constraints could be addressed by applying participatory processes in designing biosecurity measures to ensure better adaptation to local cultural and social contexts.

## Introduction

Pig production in Uganda involves predominantly very small herds kept under free-range management, tethered or more rarely, housed (1–3). More than 80% of herds consist of one to five pigs (4). Despite the small average herd size, the Ugandan pig population increased from 3,184,0000 in 2008 to 4,037,000 in 2016 (5). Uganda is reported to have the highest pork consumption in East Africa with an estimated annual per capita pork consumption of 3.4 kg (6, 7). Pig production in Africa is frequently associated with outbreaks of African swine fever (ASF) (8), endemic in Uganda (9). ASF is a viral disease of domestic pigs and European wild boar caused by African swine fever virus (ASFV), the sole member of the genus *Asfiviridae* (10). The disease is typically associated with high case fatality rates and clinical presentations such as high fever, anorexia, cyanosis, incoordination of movements and recumbency (11–13). Outbreaks of ASF are generally attributed to reducing the potential of pig production to contribute to income generation and poverty reduction (8). In Uganda outbreaks have been shown to have negative impact for smallholder farmers, with economic consequences increasing with the herd size and social effects such as failure to pay for school or public health fees being reported (3, 14–16). Transmission of ASF mainly occurs through direct and indirect contacts between naïve and infected pigs or products in the domestic pig-to-pig epidemiological cycle (8). In this cycle transmission further depends on the activities of people along the value chain (farmers, traders or middlemen, slaughter slab operators, butchers, pork restaurant operators and consumers), and thus all these actors are important for achieving disease control (17, 18). In the absence of vaccines, consistent application of biosecurity measures remains the only tool for preventing and controlling ASF (19, 20). In typical smallholder systems farm biosecurity is however generally limited or non-existent (21–24).

Previous studies in northern Uganda revealed that smallholder farmers have mostly positive attitudes towards the protective potential of biosecurity (3, 25), but invest very little of their pig farming income into it (14). Studies from both this and other contexts have further shown that smallholder farmers have complex livelihood situations with biosecurity representing just one of numerous concerns in their livestock production (25, 26). To prevent and control ASF, more information is needed about how implementation of biosecurity can be improved throughout the value chains. As a first step, this study aimed to explore biosecurity measures currently in use in the smallholder pig value chain in northern Uganda with a particular focus on the stakeholders' perceptions towards ASF prevention and control.

## Materials and Methods

### Study Area

The study was conducted in the Acholi subregion of northern Uganda. The subregion is among the poorest Uganda, partly due to a period of civil unrest between 1986 and 2006 (27). The Acholi people traditionally keep domestic animals both for livelihood requirement and as a part of their cultural identity (28). According to the Acholi cultural tradition, lack of respect for spirits is the major cause of “*gemo*” (epidemics). Many of the Acholi were internally displaced and lost their domestic animals during the period of civil unrest. Since 2006 the sub-region is under a government recovery program including restocking and promoting of pig farming. Despite frequent ASF outbreaks sustenance smallholder pig farming has become a major economic activity in the sub-region (9).

### Study Design and Participant Selection

In a qualitative interview study performed in October 2019 focus group discussions (FGD) were conducted to assess biosecurity measures currently in use, and elicit stakeholders' perception of ASF prevention and control. In the context of this study the term “perceptions” included aspects such as the practical feasibility, factors enabling or hindering implementation, and perceived protective effect of prevention and control measures.

**Figure 1:**
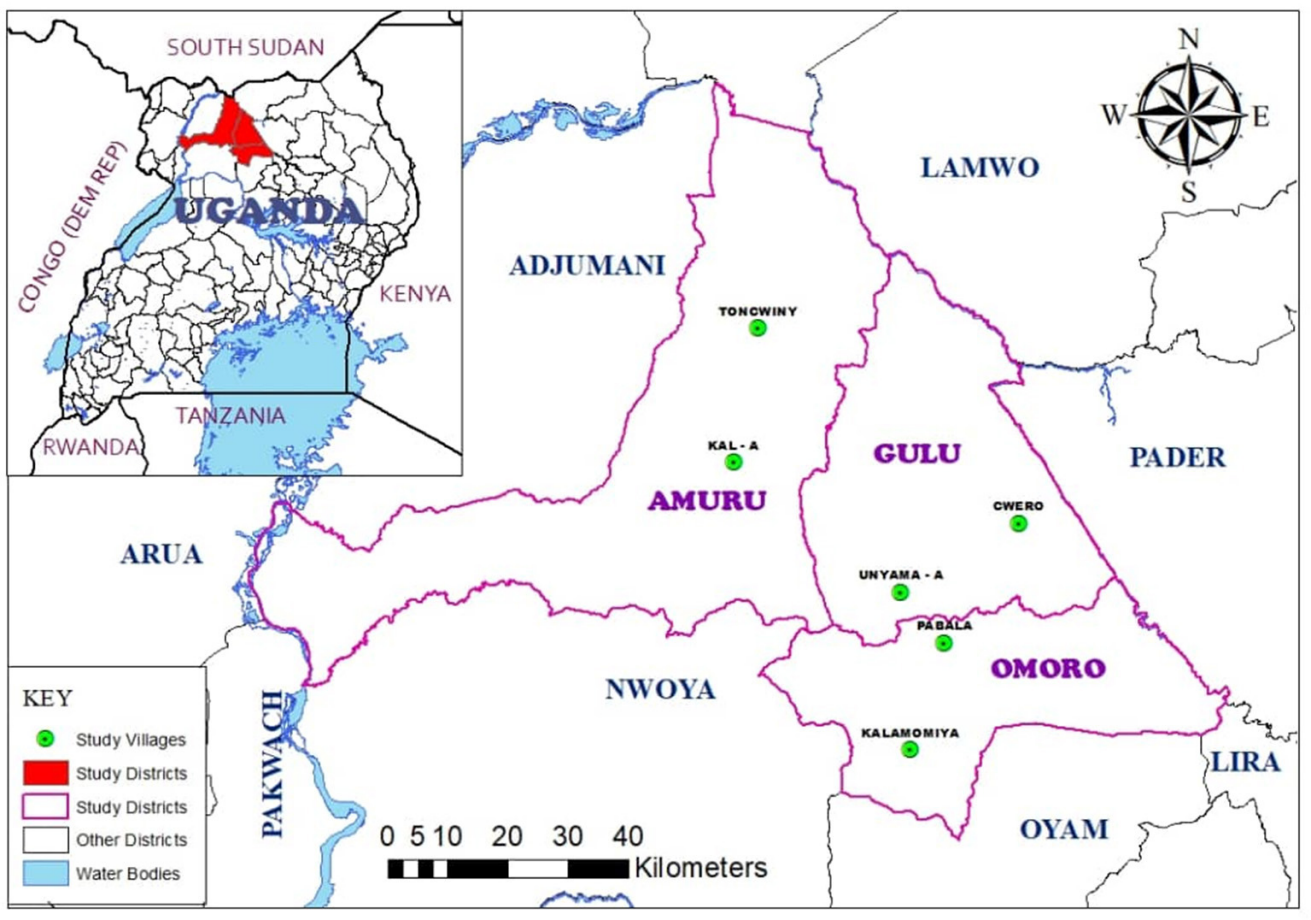
Map of Amuru, Gulu and Omoro districts where a study was conducted in northern Uganda in October 2019. Variations in geographical locations of these districts include; 02045'N, 80 32000'E (Gulu), 02035'N, 32022'E (Omoro) and 02050'N, 33005'E (Amuru).

Three districts were purposively selected based on the relative importance of pig business and from each of these two villages were included (Unyama-A and Cwero in Gulu district, Pabala and Kalamomiya in Omoro distrct, Toncwiny and Kal-A in Amuru district) (Figure 1). Inclusion of villages was primarily based on field information of suspected ASF occurrence and farmers' preliminary perceived interest in ASF control. Field information of ASF occurrence consisted of outbreak reports from district veterinary officers consistent with ASF based on clinical signs and local epidemiology. Reports were not confirmed with biological testing: based on previous participatory diseases surveillance and absence of main differential diagnoses for ASF the diagnostic accuracy of the reports was deemed sufficient for the purpose of this study (9). Participants preliminary perceived interest in ASF control was based on information from district veterinary officer and confirmed or refuted during the recruitment process. Further, the availability of a suitable number (8–12) of pig farmers for the purpose of a group discussion and known presence of several different actors along the value chain such as middlemen, slaughter slab operators, butchers, and pork-joint^1^ owners were considered in the selection. Farm or herd size was not among the inclusion criteria. Previous studies in the area show that pig production is almost exclusively performed by smallholder farmers with herd sizes ranging from 0 to 39 with an average of 3.7 pigs including piglets (14). Villages were selected by the first author in consultation with the district veterinary officers of each district. One FGD was held in each of the selected villages, hereafter identified as FGD 1 in Unyama-A village, FGD 2 in Kalamomiya village, FGD 3 in Pabala village, FGD 4 in Cwero village, FGD 5 in Kal-A village, and FGD 6 in Toncwiny village. The number of villages selected was based on the presumed number of FGDs needed to reach saturation. Participants for each FGD were further purposively selected with the intention to include as many different value chain actors as possible, and to have an equitable gender representation (Table 1). The purpose of the aspired group heterogeneity was to include a wide array of different experiences and perspectives. Participants were invited by the community animal health workers in the selected villages based on the mentioned inclusion criteria. The FGDs were conducted at venues that were convenient for participants, such as in community trading centres, sub-county headquarters, schools, health centres or the home of one of the participants. There were on average 11 participants per FGD. FGDs lasted on average 5 h 13 min (ranging from 4 h 20 min to 5 h 50 min), including a lunch break. Participants were compensated for their transport costs and lunch was provided in connexion with the interview.

**Table 1:** Distribution of focus group discussions (FGDs) participants' value chain roles and gender from a study in northern Uganda in October 2019. Traders are buyers of live pigs.

**FGD** **/value chain** **role**	**Value chain roles** ^**1**^														**Total No. of participants**
	**Purely farmer**		**Trader and** **butcher**		**Trader, butcher, and pork joint-owner**		**Trader, and pork joint-** **owner**		**Farmer, butcher and pork joint-owner**		**Farmer, trader, butcher, and pork joint-owner**		**Purely pork joint-owner**		
Gender	M	F	M	F	M	F	M	F	M	F	M	F	M	F	
FGD-1	4	4							1						9
FGD-2	4	4							3				1		12
FGD-3	7	2			1						1	1			12
FGD-4	5	3							1	1					10
FGD-5	6	2	2				1								11
FGD-6	5	3	1			3									12
Total	31	18	3		1	3	1		5	1	1	1	1		66

1 *Some participants were involved in several activities in the value chain*.

### Data Collection

The research team was composed of a facilitator (TA), a senior researcher (EC), and a translator proficient in both the local language (Acholi) and English. Both the facilitator and the translator had been trained in qualitative research approaches and the procedure for data collection prior to the study. The FGDs started with the facilitator introducing the purpose of the study, specifically emphasising that it was research and not a needs assessment or similar with possible immediate benefits for participants. It was explained that participation was voluntarily. Confidentiality was assured and permission sought to take photographs and make voice recordings, and participants signed a written consent form to this effect. The discussions which started with farmers' experience of ASF outbreaks (Table 2) were conducted in Acholi and simultaneously translated and recorded. Detailed notes were taken during the FGDs, and the recordings were subsequently transcribed verbatim. Discussions followed a topic guide (see Appendix 1) centred on the measures participants had implemented to prevent ASF outbreaks and control the spread of ASF during outbreaks. The facilitator guided the discussion according to the topic guide, while letting participants lead the discussion to subjects that were important to them.

**Table 2:** Details of recent African swine fever outbreak from a study in northern Uganda in October 2019.

**Groups**	**Last occurrence**	**Participants' comments**
FGD-1	April 2019	Common during cold weather, dry spell and beginning of rain.
FGD-2	June 2019	Serious during sunshine, and heavy rain season
FGD-3	May 2019, October 2019	Common during bad hot weather and rainy season
FGD-4	May/June 2019	During dry spell and in December when it is hot. When it's hot and a lot of wind
FGD-5	April/May 2019	When the rain starts every year. Comes during dry spell with sunshine and dust
FGD-6	April/June/Aug/2019	Outbreaks are always during dry season

*FGD, focus group discussion*.

### Data Analysis

**Figure 2:**
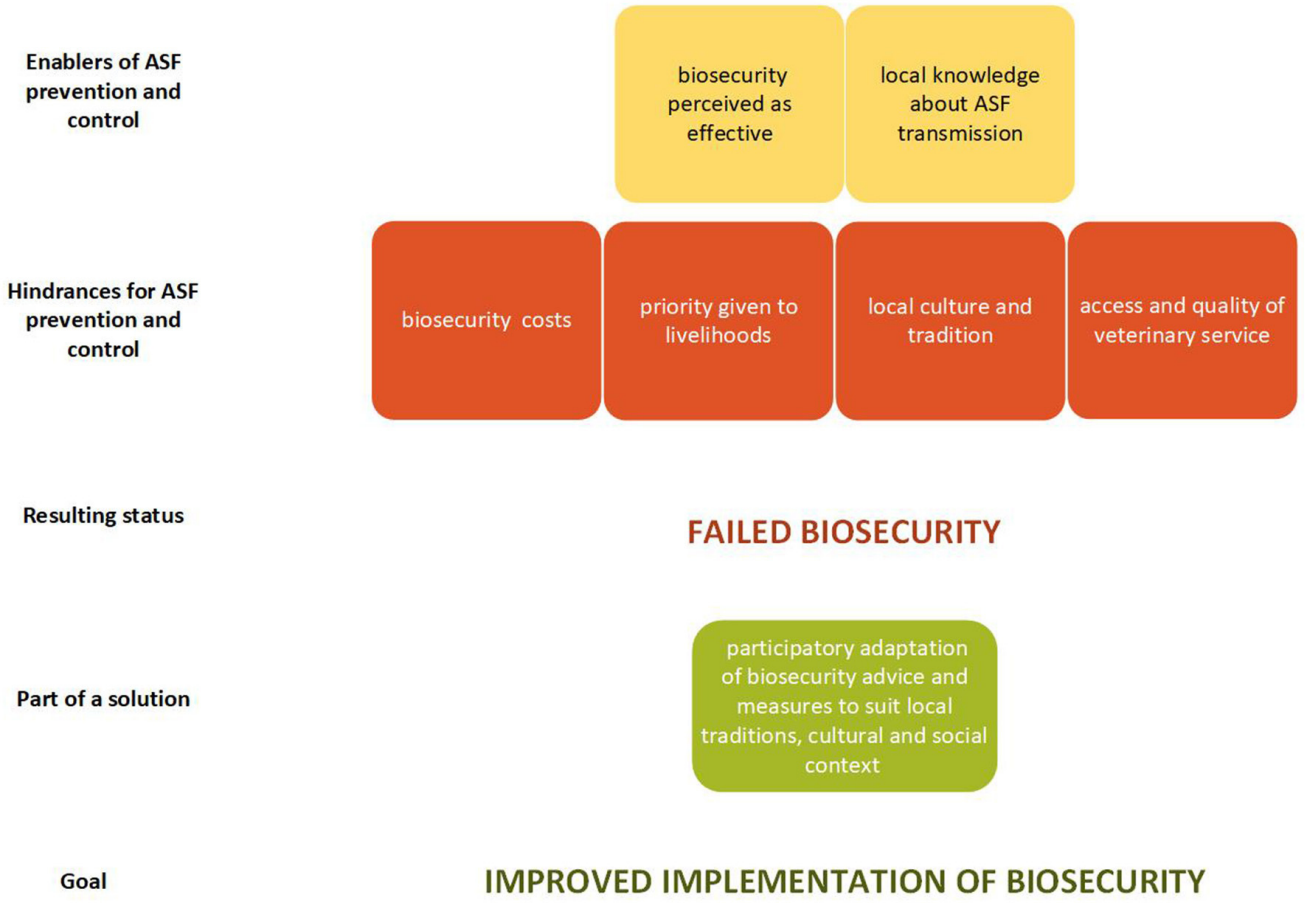
Logical framework illustrating the results from a study in northern Uganda in October 2019.

The field notes and transcripts of the audiotapes were merged to form one master set of notes for each FGD. In a first step, all mentions concerning biosecurity were identified (Table 3). Secondly, thematic analysis was applied to the master notes (29). This was done independently by two of the authors (TA and DMO). In this step, every segment of the data relevant to perceptions of prevention and control were coded to represent the initial stage in the conceptualisation of the transcribed data. These primary codes were allowed to emerge inductively through repeated reading of the data, while forming hypotheses about the data that were subsequently refined in repeated rounds of analysis, comparing and merging the two sets of codes (30). Applying axial coding, TA and DMO together sorted the primary codes into common themes (31) (see Appendix 2). Finally, TA combined themes associated with others to form six categories. The themes and categories are summarised in a matrix (Table 4) for ease of traceability back to the original data, to provide the relationship between the themes and the FGDs and to illustrate the frequency of mentions (32). The emerging categories, their internal relation, additive inference for ASF prevention and control, and a suggestion for improving implementation of biosecurity was visualised in a logical framework (Figure 2).

**Table 3:** African swine fever biosecurity measures mentioned in a study in northern Uganda in October 2019.

**Mentions of biosecurity**
**General hygiene practises:** Good hygiene practises, use of disinfectant, do not use the same equipment for pigs and at home
**Minimising indirect contacts:** Few attendants allowed in the pig house, non-attendants do not enter the pig house
**Minimising direct pig-to-pig contacts:** Build pig house, fence pig house, confinement of free-range pigs, tether pigs, stray pigs should not enter the pig house, do not borrow boars
**Feed and water of good quality and sufficient quantity:** Provide feed, avoid swill feeding, provide enough water
**Safe carcass handling:** Bury or burn carcasses
**Restriction of trade in pigs and products:** Don't bring pork home, stop buying pigs during outbreaks
**Sharing of pig health information:** Alert neighbours during outbreak, call veterinary personnel
**Other:** Remain close to pigs all the time

**Table 4:** Perceptions of African swine fever (ASF) prevention and control among pig value chain actors from a study conducted in northern Uganda in October 2019.

**Categories and themes/FGD/Number of mentions**	***FGD 1***	***FGD 2***	***FGD 3***	***FGD 4***	***FGD 5***	***FGD 6***	**Total no. mentions**
**ASF biosecurity measures perceived as effective**							
Pig can be confined in houses or by fences, and these can be constructed in different ways	✓	✓	✓	*x*	*x*	*x*	3
Confining pigs prevent contact with other pigs and people	✓	✓	✓	*x*	*x*	*x*	3
Restrict pigs' movement to control what the pigs eat and avoid contact with sick stray pigs and contaminated items	✓	✓	✓	✓	✓	✓	6
Disclosing animal health status	*x*	*x*	*x*	✓	✓	*x*	2
Implementation of local punitive measure	✓	*x*	*x*	*x*	*x*	*x*	1
**Local knowledge of ASF transmission**							
ASFV can be transmitted by the wind	✓	✓	✓	✓	✓	✓	6
Damp ASFV cannot be blown from carcasses dumped in the swamp	✓	✓	*x*	*x*	*x*	*x*	2
Flies and wind can carry infective materials	✓	✓	✓	✓	✓	✓	6
Dogs, pigs and people can bring contaminated pork or bone	✓	✓	✓	✓	✓	✓	6
Feed and water contaminated with urine, faeces and saliva	✓	✓	✓	✓	✓	✓	6
People contaminated with faeces and blood	✓	✓	✓	✓	✓	✓	6
Borrowing breeding boars for mating	*x*	*x*	✓	✓	✓	✓	4
Contaminated unwashed hands handling feed and pigs	*x*	*x*	✓	*x*	✓	*x*	2
Use of contaminated utensils, farm tools and protective gear	*x*	*x*	*x*	✓	✓	*x*	2
Middlemen and slaughterers can transmit disease	✓	✓	✓	✓	✓	✓	6
Trade in live pigs can transmit disease	✓	✓	✓	✓	✓	✓	6
Vets can transmit disease	*x*	*x*	*x*	✓	*x*	*x*	1
Cool temperatures protect pigs, heat kills ASFV	✓	✓	*x*	*x*	*x*	*x*	2
Disinfection using ash and “Jik”^1^.	*x*	*x*	✓	✓	✓	*x*	3
Basic hygiene	*x*	*x*	✓	*x*	✓	*x*	2
IMO technology adoption^2^.	*x*	*x*	*x*	*x*	✓	*x*	1
Leaving farm tools and protective gear at the pigsty	*x*	✓	✓	*x*	✓	*x*	3
Isolating sick or relocating healthy pigs	*x*	✓	*x*	*x*	✓	✓	3
Feed quality and quantity is important for good health and fast growth	*x*	✓	✓	*x*	*x*	*x*	2
**Implementation of biosecurity is partially hindered by cost**							
Disinfectants, cleaning materials, building materials, fuel and feeds are unaffordable	✓	✓	✓	✓	✓	✓	6
**Priority given to livelihoods**							
Carcasses are consumed at home or sold to raise some money and avoid total losses	✓	✓	✓	✓	✓	✓	6
Trade in live pigs to protect healthy ones, raise some money and avoid total losses	*x*	✓	✓	*x*	✓	✓	4
People bring pork home to eat	✓	✓	✓	✓	✓	✓	6
Butchers and middlemen make a profit during outbreaks	✓	✓	✓	✓	✓	✓	6
Selling sick pigs poses risk of ASF spread	✓	✓	✓	✓	✓	✓	6
**Local culture and traditions**							
Burial of animals is forbidden in the Acholi culture and tradition	*x*	*x*	✓	✓	✓	✓	4
It is hard work to dig a grave	*x*	*x*	*x*	*x*	✓	*x*	1
It is psychologically painful because it reminds you of burying loved ones	*x*	*x*	*x*	✓	*x*	✓	2
People can throw bones, pork, and intestine in the pigsty to intentionally infect healthy pigs	*x*	✓	✓	✓	✓	✓	5
**Access and quality of veterinary services**							
Smallholder farmers have access to veterinarians	✓	*x*	✓	✓	✓	✓	5
Smallholder farmers do not have access to veterinarians	✓	✓	✓	✓	*x*	*x*	4
Veterinary treatments are helping	✓	*x*	✓	✓	*x*	✓	4
Veterinary treatments are not helping	✓	✓	✓	✓	✓	*x*	6
There is no medicine or vaccine for ASF	*x*	✓	✓	✓	✓	✓	5

*ASFV, African swine fever virus; FGD, focus group discussion; IMO, indigenous microorganisms*.

***Categories are written in bold on a grey background, themes belonging to each category are listed underneath***.

*✓ = theme was present in this focus group*

*✗ = theme was not present in this focus group*

1 *Ash (residue after burning materials) are poured at door entrance to replace footbath. “Jik” is the trade name of a detergent*.

2 *Indigenous microorganism e.g. “lactic acid bacteria” trapped in a solution are poured on floor of pigsty to decompose pig faeces*.

## Results

### Participant Characteristics

A total of 65 participants (between nine and twelve per FGD) were included in the six FGDs (Table 1). Out of these, 48 were purely pig farmers and 17 were involved in different business along the value chain (some of these participants also kept pigs but did not consider pig farming their main occupation). Pig farmers had between three and six pigs each.

### Experience of ASF Outbreaks

Recent ASF outbreaks were mentioned to have occurred in the months of April, May, June, August and October 2019, during both dry and wet season (Table 2). Sudden death, change in body colour displayed as red ears and hooves, reduced appetite, difficulty in walking and dullness were each mentioned by at least two FGDs as clinical signs suggestive of ASF. FGDS 2, 4, and 5 reported massive pig deaths as the determinant sign of ASF.

### ASF Biosecurity Measures

During the FGDs, 20 different biosecurity measures were mentioned. Many of these were similar and seven groups of more general biosecurity or husbandry measures could be identified (Table 3).

### Thematic Analysis of Perceptions of ASF Prevention and Control

During the thematic analysis, 39 themes were identified from the emerging primary codes. The themes were summarised into six categories (Table 4 and Appendix 2). The categories included “ASF biosecurity measures perceived as effective,” “local knowledge of ASF transmission,” “implementation of biosecurity is partially hindered by its cost,” “priority given to livelihoods,” “local culture and traditions,” and “access and quality of veterinary services.” Narrative details of themes and categories are explored below.

### ASF Biosecurity Measures Perceived as Effective

Biosecurity measures were generally perceived positively by participants. Construction of pigsties or fences were often mentioned as helpful for preventing contact between confined and free-range pigs, and with people. Participants in all FGDs stated that confined pigs are protected from coming into contact with infective materials (e.g., bone and pork) or contaminated water in the environment: “*Pigs confined in the pigsty survived; pigs that were released to be on free range contracted ASF and all died”* (FGD 3). Sharing information about ASF outbreaks was also seen as helpful, serving to alert farmers to improve biosecurity measures and to take protective measures if, for example, pork was brought home for consumption. Farm-gate buying and selling of live pigs was mentioned as a common practise. Participants in one FGD said that they would welcome stricter implementation of punitive measures set locally to improve biosecurity, such as obligatory confinement of pigs and trade restrictions concerning live pigs and pork if outbreaks have been recorded in the area. In this regard, participants said that people did not fully comply with existing by-laws: “*Some people follow the rules, but others don't”* (FGD 1).

### Local Knowledge of ASF Transmission

Participants in all FGDs perceived that airborne transmission of ASFV could occur if the virus from infective tissues or ashes from burned carcasses was carried by wind. Similarly, participants stated that flies can be contaminated with ASFV while feeding on infective materials and then transmit the virus to feed or water, which in turn can infect healthy pigs. In all FGDs, participants mentioned that vegetables and water could be contaminated with saliva, faeces or urine from infected stray pigs and serve as a source of infection if served to healthy, confined pigs. Participants further said that faecal matter and urine excreted by infected confined pigs could contaminate feed left on the floor or in the feed trough. It was mentioned that infective remains of partly burned carcasses could be eaten by free-range pigs. In this regard, participants also said that pigs could dig up carcasses if they were not properly buried. In some FGDs, participants said that people who buy and bring pork home could infect healthy free-roaming pigs if bones were not discarded out of the pigs' reach.

Participants in all FGDs mentioned that confined pigs could become infected if butchers and middlemen wearing blood-stained clothes were allowed to enter the pigsty and touch pigs before purchase: “*Farmers would see money and just let the butcher enter the pigsty”* (FGD 2). Participants further said that stray boars that enter pigsties (either on their own or being driven into the house) to mate with sows could transmit ASFV. In some FGDs participants said that ASF transmission could occur if people who had handled infected pork subsequently handled pig feeds without first washing their hands. Likewise, they said that saucepans used to carry pork from the market and subsequently used to water pigs without prior washing could transmit ASF. They also described how ASF could be transmitted if the same wheelbarrow was used to carry contaminated maize from the fields and for pig feeds. Having clothes, gumboots and wheelbarrows strictly for use in the pigsty was mentioned as a good practise that could stop the spread of ASF. The practise of disinfecting shoes at the door and washing hands before entering the pigsty would still leave other body parts contaminated: “*Chemicals will kill the virus on gumboots and shoes, but not on hair or clothes”* (FGD 3).

Avoiding purchasing pigs during ASF outbreaks was mentioned as a way of preventing disease transmission because sick or in-contact pigs are frequently marketed: “*Farmers sell sick pigs cheaply without disclosing their health status to buyers”* and “*Buyers know sick pigs by their low price”* (both FGD 3).

Participants mentioned “cool temperature” as one factor that could influence ASF transmission. According to their described experience, healthy pigs that were relocated to swampy areas during an ASF outbreak would not die. Meanwhile, sick pigs left on the farm would die of the fever, accelerated in their view, by high temperatures in the pig house. They further described how the wind would not transmit ASFV from carcasses if they were dumped in swampy areas: “*It is better to throw it in the water so that the virus is not blown by the wind”* (FGD 5). Farmers noted that staying close to pigs to immediately remove faeces could serve to maintain good hygiene, and that this could prevent the spread of ASF. Participants in one FGD mentioned that adoption of indigenous microorganism (IMO) technology for floor bedding (using rice bran) in the pigsty could decompose faeces and urine, serving the same purpose. Participants also said that the isolation of sick pigs prevented healthy pigs from succumbing to ASF. Among the different modes of isolation, participants mentioned transferring/relocating healthy pigs to disease-free villages. Provision of good-quality feeds in sufficient quantity was mentioned as boosting the immunity of pigs and stimulating fast growth. The latter was specifically said to be useful for getting pigs to market size before the onset of anticipated ASF outbreaks. ASF outbreaks was mentioned to occur throughout the year: “*We believe that it is brought by hot weather”* and “*If it is serious sunshine, the outbreaks get serious”* (both FGD 2).

### Implementation of Biosecurity is Partially Hindered by Its Cost

Participants revealed that many community members could not afford to buy pig feeds or disinfectants. In addition, commercial feeds (e.g., maize and rice bran) were mentioned as being difficult to access. Farmers who could not afford to buy feed said that they let pigs roam free to scavenge, and that supplementary feeding was done using swill collected for free from restaurants or home kitchens. Participants frequently mentioned that it would be too costly to use fuel to burn carcasses, and that dead pigs need to be marketed (and not destroyed) to recover as much of the incurred losses as possible. Some farmers mentioned that they manage to prioritise investing in biosecurity and buying materials for constructing and cleaning the pigsty.

### Priority Given to Livelihoods

Participants said that they slaughtered sick pigs for home consumption, sold pork from pigs that had died to neighbouring communities to raise money, and used pork to barter for other food items as a way of coping with the hardship experienced during ASF outbreaks: “*When you have a pig sick with ASF and don't want others to know, you slaughter it and sell it quickly, and say that the pig was strangled by the tethering rope”* (FGD 4). Participants also said that sick pigs were sold to protect the health of the remaining pigs, to raise money and to avoid the total loss of investments that would occur if the pigs succumbed to ASF. Healthy pigs that had been in contact with suspected ASF cases were reportedly sold for the same reasons and with the aim to use the earnings to restock pigs after the outbreak. As pork is cheaper than usual during outbreaks, participants said that people would take these opportunities to buy pork and bring it home for family consumption or go to eat at places such as pork joints and hotels^2^. Participants further reported that whenever pigs are slaughtered, neighbours not rearing pigs might buy pork and take it home to eat. Farmers reported that middlemen and butchers would buy pigs (sick and healthy) at a low price during ASF outbreaks, but maintain the regular sale price for both fresh and cooked/roasted pork, thus taking advantage of a low buying price to make a profit.

### Local Culture and Traditions

Participants in several FGDs mentioned that, according to the Acholi culture and tradition, the burial of animals is forbidden. They said that if animals were buried, other animals would die. In some FGDs, participants said that all animals except dogs could be buried without there being bad consequences, whereas burying dogs would be associated with the disappearance of rain for a year. Apart from the cultural taboo related to the actual act of burying animal carcasses, frequent mention was made of contextual constraints regarding throwing away food, and in particular meat, in the poor study communities. Repeated mention was also made of people possibly digging up buried carcasses to get access to the meat. In addition, they said that burying animals is painful because it reminds them of burying loved ones. The hard work required to dig a hole deep enough to prevent carcasses being exhumed by animals or people was mentioned as discouraging the implementation of this practise for safe carcass disposal. All FGDs mentioned that people (in most cases neighbours) sometimes threw bones, pork or intestine in other peoples' compounds to infect pigs deliberately out of jealousy. This was exemplified as follows: “*Poisoning of pigs in our area by people who are jealous has become a culture”* (FGD 4) and “*When I saw somebody dropping pig bones in my pigsty, I was furious. I immediately let all the pigs out, and they all ran to the swampy area. Some pigs died, others survived”* (FGD 3).

### Access and Quality of Veterinary Services

Smallholder farmers in four FGDs expressed their trust in local veterinarians for the diagnosis of ASF as well as for giving advice on disinfection procedures and other prevention practises. In the study area, veterinarians qualified to diagnose and treat animals can be either employed by the government or private. Participants mentioned that veterinarians were generally available, and they were praised for giving advice to farmers on how to manage pig health during outbreaks. However, three FGDs had generally negative accounts, mentioning that veterinarians frequently either did not respond to farmers' calls, or responded so late that all the pigs they treated ended up dying: “*Veterinarians say there are no drugs, so won't respond to farmers' calls”* (FGD 4). The negative accounts further included mention of veterinarians offering generally bad services and giving false expectations about the survival possibilities of ASF-infected pigs if treated: “*Veterinarians injected pigs, the medicine didn't work, all the treated pigs died”* (FGD 5). In this regard they also mentioned some veterinarians asking for payment from farmers for treatment, even if there is no hope of saving the pigs. In one FGD, farmers noted that veterinarians could play a role in transmitting ASF as they reuse needles between different herds. These accounts also included complaints about veterinarians not making farmers aware of the dangers of ASF.

Participants in five FGDs mentioned that they had no hope of saving the lives of pigs infected with ASF, and that the disease has no treatment or vaccine, unlike other pig diseases that can be cured if sick pigs are treated: “*Farmers vaccinate*^3^ *against other diseases such as flu and worms” (FGD 4), “The vaccination has no effect on ASF”* (FGD 2). Farmers said that they do not call veterinarians to treat pigs for ASF as there is no cure for it: “*Veterinarians would say there is no drug for ASF, so they would not come to treat it”* and “*It's not easy, some pigs could still die even if the vet treated them”* (FGD 3).

### Logical Framework

The emerging categories encompassed both “enablers” and “hindrances” for ASF prevention and control (Figure 2). Participants mostly had positive perceptions of ASF biosecurity, describing many measures as feasible to implement and effective for preventing or controlling ASF outbreaks. Together with rich local knowledge of ASF transmission this enabled prevention and control of ASF. Some of the knowledge corresponded to current scientific understanding and practises and some not. Misbeliefs regarding aerial (air-borne) transmission or virus survival in ash might hamper effective implementation of biosecurity, whereas knowledge regarding transmission *via* direct and indirect contacts facilitated achieving control. Four categories were seen as hindrances. Participants' efforts to implement ASF prevention and control measures were limited by: biosecurity costs such as building material and pig feeds that is necessary if pigs are to be confined; the need to prioritise family livelihood over known disease transmission risks connected to for example trade in sick pigs or carcasses from pigs that have died from disease that could be ASF; local culture, traditions and social factors that e.g., complicated safe destruction of carcasses; and finally lack of access to veterinarians or, occasionally, due to low-quality veterinary services. Together this often resulted in failed biosecurity, with the main hindrances seemingly being the cost of the measures, and to some extent the incompatibility of current biosecurity practises with local traditions, context and culture. Both these constraints could be addressed by adapting biosecurity measures to local cultural, social and economic contexts in a participatory process involving the concerned end users.

## Discussion

This study showed that perceptions of biosecurity in the smallholder pig value chain in northern Uganda were mostly positive, especially regarding the preventive effect of rather simple measures such as confining pigs and not buying pork during outbreaks. The study did not seek to assess participants' knowledge of ASF, but still captured rich accounts of local knowledge about ASF transmission. It therefore appeared as if neither participants' perception of biosecurity nor their knowledge were the main limiting factors for implementation of ASF biosecurity, in accordance with previous studies from similar settings in Uganda (3, 33).

Health behaviour models have been used to describe and increase the understanding of people's behaviour and decision making in relation to their own or their animals health or disease risks (34). Many such models refer to social cognitive behavioural theory to explain behaviour and decision making, in short attributing the factors determining behaviour to the characteristics of the individuals and their social networks (35–38). The categories emerging as enablers of implementation of biosecurity in this study (“ASF biosecurity measures perceived as effective” and “local knowledge of ASF transmission”) could be considered as social cognitive factors, relating to beliefs, attitudes or knowledge (39). Categories hindering implementation of biosecurity (“implementation of biosecurity is partially hindered by its cost,” “priority given to livelihoods,” “local culture and traditions,” and “access and quality of veterinary services”) however, were linked to contextual and social factors (40). Ebata et al. (40) describe how such contextual factors can make compliance difficult or even impossible for local people. According to the results, costs of constructing houses or enclosures and providing feed if pigs are prevented from scavenging were in many cases perceived as unaffordable, and could thus not be implemented despite the positive perceptions of these measures. Similar reasons for the failure to implement the most basic biosecurity measures necessary for minimising direct and indirect contacts have previously been reported (16, 33, 41). Likewise, minimal investments in pig feeds have previously been reported from the same area (14), with pig diets consisting of vegetables, cassava peelings and swill, or pigs scavenging for food. Swill feeding, which is continuously practised as a way of reducing feed costs, is a risk factor for ASF management, especially in endemic areas (3, 22, 42). Affordability of inputs and measures that are developed and adapted with end users, thus ensuring suitability and local acceptance, have been suggested as key to achieving functional biosecurity (43). The term “functional biosecurity” is used here to indicate that it is the operational end-result of the total biosecurity efforts, that sufficient biosecurity is always implemented at all risk activities, that is the most important aspect of biosecurity. The opposite, failed biosecurity, have been attributed to lack of biosecurity routines or equipment (biosecurity hardware), or routines that are prescribed but not implemented (biosecurity software) (44).

Providing for the family livelihood and sustaining income were clear priorities for the participants, overriding other concerns such as the risk of transmitting disease or not complying with regulations such as trade restrictions in connexion with ASF outbreaks. The high poverty levels in the study area, among the highest in Uganda (45), most probably contribute to the priority given to sustaining income. In the study area, pigs are mostly not kept for household consumption, but mainly to provide money for school fees or unforeseen healthcare events, and to barter for agricultural labour (14). Consequently, the sale and slaughter of clinically sick or in-contact pigs is practised (14, 40, 46) in order to retain the benefits from this resource, support household livelihoods and avoid financial loss. This coping mechanism was reported in this study. Participants also described how traders and butchers buy pigs at lower prices during ASF outbreaks but sell at normal prices, hence making greater profits and possibly contributing to ASF transmission while travelling to affected villages in search of cheap pigs.

The study noted how the lack of adaption of biosecurity measures to local culture and traditions resulted in failed implementation. Specifically, this concerned the disposal of carcasses from pigs that have died from the disease as a taboo linked to misfortunes, which were frequently reported in connexion with the burial of animal carcasses. In addition to being an important protein source in the food-insecure study area, the local communities considered meat a delicacy and, as such, it is unacceptable to bury or dispose of meat by burning. Consequently, a willingness to buy or barter pork from dead pigs was reported. The opportunity to make money from diseased or dead pigs serves as a disincentive to dispose of carcasses (3, 14, 47). Additionally, gathering to buy or barter pork from dead pigs allows community members to maintain social capital around an otherwise mere catastrophic event. In both these examples, pork serves as a social and economic resource that concurrently carries the negative attribute of possible ASF transmission. It was reported that carcasses disposed of by burial were exhumed for consumption. Other means of carcass disposal or virus inactivation that fitted better with local culture and traditions (such as heating or drying out of the reach of pigs) are thus needed to achieve functional biosecurity in this context. (40) Ebata et al. (40) discuss how farmers are hindered from investing in biosecurity by contextual or structural factors such as poverty. Likewise, in this study, resource constraints as well as cultural and traditional factors seemed to influence participants' opportunities to improve implementation of biosecurity. As an example of how implementation of biosecurity can be improved in resource poor settings and in agreement with local culture and traditions, a study from Timor-Leste report that participatory adaptation of biosecurity measures to the local context and applying methods inspiring community commitment motivated changes in pig management preventing ASF outbreak in study villages (48, 49).

Finally, the results suggested that access to veterinary health care was limited and hindered ASF prevention and control for some participants. In addition, the professional relationship between local veterinarians and farmers was complicated by suspicions that veterinarians' lack of clinical hygiene might contribute to the spread of ASF. Wesonga et al. (50) conclude that animal disease management in Uganda is ineffective, and that this is associated with inadequate and inefficient delivery of even the most basic, mandatory veterinary services.

There is no evidence supporting aerial (air-borne) transmissions of ASFV for more than a few metres inside and around pig pens (51, 52). According to local knowledge, however, ASFV was frequently mentioned to be transmitted aerially, and ashes of burnt pig carcasses mentioned as a risk factor for transmission. Farmers who perceive ASF as air-borne are unlikely to implement biosecurity measures that could prevent introduction of ASF, as these will not be perceived as effective if the virus “flies in the air.” Likewise, an effective way of eliminating ASFV by burning carcasses at temperatures higher than 60°C (53, 54) will not be performed if the ashes are considered infective. In this regard education actions targeting specific epidemiological subjects of concern might improve implementation of biosecurity.

The study design included purposive selection of study sites and participants. This ensures that the results are important for the local context but limits how the results can be extrapolated to other contexts. In this regard the selected farmers were considered to provide a fair representation of the study population in terms of herd size and pig management. Recent ASF outbreaks was reported from all villages, no further information regarding these outbreaks was however collected meaning that associations between individual responses regarding perceptions of ASF prevention and control and experiences of ASF outbreak could not be investigated. Equal gender representation was not achieved in this study. Although men make most decisions concerning resource allocation for biosecurity, pigs are mostly managed by women (4). The underrepresentation of women could thus have led to selection bias in this regard. FGDs were held in Acholi but the analysis was made from transcripts translated to English. This could have led to loss of information depth (55). In this study this risk was reduced as the first author speaks both Acholi and English. Aspects of hidden and open power dynamics affecting how people can express their opinion and share experiences are present in all groups and will impact on study results (56, 57). Common ways to minimise this bias is to aspire that groups are as homogenous as possible regarding i.e. gender, occupation or poverty level, and not seek consensus but encourage diversity (58). In this study efforts were made to record all opinions and consensus were not sought. Frequency of mentions were however recorded, with themes that were more present than others in the data given more weight in the final (qualitative) analysis.

In conclusion this study demonstrated that despite mostly positive perceptions of biosecurity, biosecurity measures were not being implemented due to costs of feed and housing, and the fact that family livelihood had to be prioritised over investments in disease control. Other hindrances were limitation in veterinary access and quality of services, and biosecurity measures that were not adapted to local culture and traditions. Achieving functional ASF prevention and control thus seems to require careful adaption of biosecurity advice in participation with end users, taking local traditions, culture and the socioeconomic context into consideration. Access to pig feed and quality veterinary services are aspects that need attention in this regard. The inclusion of local veterinarians in participatory discussions on biosecurity and herd health could strengthen the client-veterinary link and improve veterinary access.

## Data Availability Statement

The raw data supporting the conclusions of this article will be made available by the authors without undue reservation.

## Ethics Statement

The studies involving human participants were reviewed and approved by School of Health Sciences Research and Ethics Committee, Makerere University Ref. No. 2019-062. The patients/participants provided their written informed consent to participate in this study.

## Author Contributions

This study was designed by EC and KS. EC and TA performed the fieldwork. TA and DMO conducted the thematic analysis. TA drafted the manuscript. EC interpreted and conceptualised the results. All authors revised and approved the final manuscript.

## Funding

This study was carried out with financial support from the Swedish Research Council VR (ASF-Implement: contract no. 2017-05518), African Union research grants (ASF-RESIST: contract no. AURG II-1-196-2016) and Gulu University (ADB-HEST: contract no. P-UG-IAD-001).

## Conflict of Interest

The authors declare that the research was conducted in the absence of any commercial or financial relationships that could be construed as a potential conflict of interest.

## Publisher's Note

All claims expressed in this article are solely those of the authors and do not necessarily represent those of their affiliated organizations, or those of the publisher, the editors and the reviewers. Any product that may be evaluated in this article, or claim that may be made by its manufacturer, is not guaranteed or endorsed by the publisher.

## Footnotes

^1^“Pork-joints” are small kiosk-like restaurants serving only pork.

^2^“Hotel” is the name used in Ugandan English for restaurants serving different sorts of meat.

^3^It should be noted that in the local context “vaccinate” might refer to both preventive and curative injections. This was not further investigated here.
