# Supplementary material for: Prevention and Control of African Swine Fever in the Smallholder Pig Value Chain in Northern Uganda: Thematic Analysis of Stakeholders' Perceptions
Source: Front Vet Sci. 2022 Jan 13;8:707819. doi: 10.3389/fvets.2021.707819 (PMC8793068; doi:10.3389/fvets.2021.707819)
Supplement: Supplementary file 1 [file Data_Sheet_1.doc]

**APPENDICES**

**Appendix 1: Focus group discussion topic guide**

***Outline***

1. Present the project, its objectives and the project team
2. Explain the consent form and confidentiality, ask for permission to take photographs and make recordings
3. Complete the background data sheet and consent forms and pay the transport fee
4. Start the discussion

***Material needed***

Background data sheet, flipchart, scotch tape, markers, notebook, pen, digital recorder, refreshments

***Time started _______________Time ended _________________***

***Interview/topic guide***

***Warm-up***

Have you or the village been affected by African swine fever?

When was the last time this happened?

Is there anything you would like to say about the outbreak(s)?

We are going to talk a bit about today what you did before and during that outbreak.

***Control and prevention***

Did/do you try to prevent ASF from entering your village?

How did you do that?

*(list all the prevention measures mentioned on the flipchart)*

What worked well and why?

What didn’t work well and why?

When there was African swine fever in the village, what did you do?

*(list all the control measures mentioned on the flipchart)*

What worked well and why?

What didn’t work well and why?

**Appendix 2: Coding of transcripts from focus group discussion in a study conducted in northern Uganda in 2019.**

| **Extracts from transcripts** | **Primary codes** | **Themes** | **Categories** |
| --- | --- | --- | --- |
| Houses have different standards  Survival of pigs depends on house standards  Houses should have a fence  Fences stop stray pigs  House with compartments  Wide gaps in the wall can allow stray pigs to meet indoor pigs nose to nose | Biosecurity measures | Pigs can be confined in houses or by fences, and these can be constructed in different ways. | ASF biosecurity measures perceived as effective |
| Pigs should not be released from their houses when feed is scarce  Other people won’t touch pigs  People who step in faeces don’t enter | Biosecurity measures | Confining pigs prevents contact with other pigs and people |
| Pigs moving around  Movement restrictions  Movement restrictions  Restrict movement so infected bones aren’t eaten  Pigs move to eat  Moving stray pigs can be contaminated  Stray pigs cannot be chased at night when you’re asleep  Movement of stray pigs  No mixing with stray pigs  Stray pigs meet with tethered pigs  They should not meet sick stray pigs  Pigs do not move from the house to come into contact with saliva & froth from sick pigs  Pigs move outside to eat anything  Tethered pigs drink running water  Not tempted to move if fed well  Stops pigs from eating swill  Satisfied pigs don’t move | Biosecurity measures | Restrict pig movements to control what the pigs eat, and avoid contact with stray pigs and contaminated items |
| Health status not disclosed  Alerting neighbouring pig farmers to improve pig management and reduce frequency of eating pork during ASF outbreak | Biosecurity measures | Disclosing animal health status |
| Have local bylaws | Laws should be used to improve biosecurity implementation | Implementation of local punitive measure |
| ASF is airborne  ASF is airborne  ASF is airborne  Wind will blow ASF into your home without you knowing | Airborne | ASFV can be transmitted by the wind | Local knowledge of ASF transmission |
| Wind will not blow ASFV from carcasses dumped in the swamp | Temperature | Damp ASFV cannot be blown from carcasses dumped in the swamp |
| Ash of burnt carcasses can transmit ASF  Flies from dumped carcasses might fly back to the house | Transmission sources | Flies and wind can carry infective materials |
| Contaminated bones  Dogs bring home the contaminated bones of unburied pigs  Pigs can access and eat the incompletely burned carcasses  Pigs and dogs can dig up buried carcasses  You can bring home pork and throw out leftovers  Children eat & throw bones around  People can throw out offal  ASF can be avoided if you don’t bring pork home  Unknowingly buy infected pork | Transmission sources | Dogs, pigs and people can bring contaminated pork or bones |
| Don’t feed on leaves to eliminate risk of spread from urine and faeces  Sweet potatoes leaves contaminated with urine and faeces can be fed to pigs  Bringing leaves contaminated with saliva and urine  Stray pigs contaminate nearby swamp water  Contaminated running water  Stray pigs contaminate water & feeds  You bring contaminated feeds into the house  Faeces can contaminate feeds in sleeping area  You can bring contaminated feeds | Transmission sources | Feed and water contaminated with urine, faeces and saliva |
| People step on faeces and enter the house  Faeces on their feet  Uncleaned faeces cause pigs problems  People step on blood  People can touch your pigs  People enter the house | Transmission sources | People contaminated with faeces and blood |
| Borrowing boars  Boars run away to mate  Boars move within villages  Stray boars allowed to mate with your pigs | Transmission sources | Borrowing breeding boar for mating |
| Eat pork from centre & prepare feeds without washing hands  Handling pigs and not washing hands  Handle pork and feed pigs with unwashed hands  Not properly washed  You wash hands & give water to pigs | Transmission sources | Contaminated unwashed hands handling feeds and pigs |
| Wheelbarrows carry maize contaminated with urine or saliva  Wheelbarrows used in the garden at home and in the pig unit  Borrowed saucepan carried pork  Use the same saucepan to carry pork and water for pig  Unclean water & bucket make pigs sick  Use cloth and leave in pig house | Transmission sources | Use of contaminated utensils, farm tools and protective gear |
| Butcher moves around villages  Butcher moves around villages using the same cloth  Butcher moves with blood on his body  Butcher slaughters sick pigs  Contamination from slaughter place  Might touch sick pigs without buying | Transmission sources | Middlemen and slaughterers can transmit disease |
| Don’t buy pigs from outside to keep yours healthy  Can’t sell piglets to neighbours from whom you can buy piglets after outbreak | Transmission sources | Trade in live pigs can transmit the disease |
| Vets might have sick pigs  Vets reuse contaminated needles | Blaming vets | Vets can transmit |
| Keep pigs cool to stop them dying  Healthy pigs survive in cool swamp  High temperature in the house kills pigs | Temperature | Cool temperatures protect pigs, heat kills ASFV |
| Footbath kills virus on the boots not hair and cloth  Use ash on doorstep to disinfect  Washing hands and legs with jik kills bacteria | Biosecurity measures | Disinfection using ash and jik |
| You can maintain good hygiene  Staying close to remove faeces immediately | Biosecurity measures | Basic hygiene |
| IMO adoption that destroys faeces and urine | Biosecurity measures | IMO technology adoption |
| Put on boots and leave at site  Have gumboots and wash house  Use of wheelbarrows & spade | Biosecurity measures | Leaving farm tools and protective gear at the pig stables |
| Isolation of sick pig that was with the rest  Isolation, healthy survive, sick die  Tie a sick pig under a tree far away  Relocation of healthy pigs  Pigs in the same compartment all die  Late isolation of sick pigs can’t guarantee the health of the remaining pigs | Biosecurity measures | Isolating sick or relocating healthy pigs |
| Pigs fed on a balanced diet fight all disease  Eat clean feed, no disease  Eat clean feed inside  Enough feeds all year round  Keep pigs without buying feed  Pigs survive on leaves  Pigs fed on leaves alone don’t grow | Feeds | Feed quality and quantity is important for good health and fast growth |
| Poverty  Unaffordable disinfectant  Expensive to buy fuel for burning  Expensive to buy paraffin  Burning requires firewood or petrol  Expensive fuel for burning  Affordability of building materials  Some pig houses are expensive to build  Insufficient money to clean house  You give free swill to pigs  Taking swill from kitchen to pig house  Financial constraints with feed  Difficult to get feed  Poor people can’t buy feed  Pigs are left free range due to unaffordable feed  Poor people cannot afford disinfectant  Difficult to buy disinfectants | Costs hinder implementation of biosecurity | Disinfectants, cleaning materials, building materials, fuel, and feed are unaffordable | Implementation of biosecurity is partially hindered by cost |
| Slaughter to eat  Slaughter the sick pig and give to children  Slaughter in the village during outbreak  People sell pork in the community  Sell pork to avoid losses  People don’t throw away adult pigs  Maybe piglet carcasses are dumped  Dead pigs have not been buried | Coping mechanisms | Carcasses are consumed at home or sold to raise some money and avoid total losses | Priority given to livelihoods |
| Sell sick pigs so as not to contaminate the rest  Farmers sell when pigs are sick  Selling sick pigs transfers ASF  Sell to get money  Sell to get money  Sell to recover money  People sell to get money  Sell mature pigs to keep money  Farmers sell to get some money  Money lets butcher enter the house  Poverty make people sell to get something  Sell the healthy pigs during outbreak  Pigs are sold before outbreak  Later buy survivors from neighbours | Coping mechanisms | Trade in live pigs to protect the healthy ones, raise some money and avoid total losses |
| People like pork  Everybody likes meat  People need to eat meat  You need to eat pork  People will eat buried pork  Eat from the hotel  People eat contaminated pork  I might not buy pork but neighbours can bring pork home  People come & buy meat  People are buying pork  People buy pork from outside & bring it home  People buy this pork | Pork | People bring pork home to eat |
| Butchers buy pigs  Sick pigs bought by butchers can die before reaching the slab  Butchers buy cheap pigs during outbreak  Butchers buy cheap sick pigs  Hard for a butcher to identify sick pigs  Butchers & middle men buy sick pigs | Blaming butchers | Butchers and middlemen make a profit during outbreak |
| Selling sick pigs transfers ASF  Buyers can bring in disease on their contaminated clothes  Buy sick pigs unknowingly  Buying pigs from unknown source | Transmission sources | Selling sick pigs poses risk of ASF spread |
| Some people accept bringing swill  People don’t bury  Acholi culture doesn’t accept burial  Other animals can be buried, but not dogs  Burial is bad in the African tradition  Culture doesn’t allow burial, taboo  Other animals will die  Rains can disappear for a year when dogs are buried | Burial | Burial of animals is forbidden in the Acholi culture and tradition | Local culture and traditions |
| Digging a grave is hard | Burial | It is hard work to dig a grave |
| Burial is painful  Like burying a human being  Stray pigs or people might be exhumed if not well buried | Burial | It is psychologically painful because it reminds you of burying loved ones |
| Jealous people throw bones in the house  Jealous people can throw bones  Jealous people throw bones and pork  People can throw carcasses  Neighbours buy and throw bones  Stop people from throwing bad things  Intestines thrown to dogs  Someone can bring infected bones after a large pig has been sold | Jealousy | People can throw bones, pork, and intestines in the house to deliberately infect your pigs |
| You follow the vet’s advice  Vets give advice to avoid ASF | Trusting vets | Smallholder farmers have access to a vet | Access and quality of veterinary services |
| Vets don’t come out to treat ASF  Vets don’t make farmers aware of it | Blaming vets | Smallholder farmers don’t have access to vets |
| Vets advise on disinfection before entering the house  Vets diagnose the disease  Vets are accessible by smallholder farmers |  | Veterinary treatments are helping |
| Pigs still die after the vet is called  Vets treat the pig and it dies if it has ASF  Vaccination doesn’t help  Calling a vet doesn’t help  Vets give the wrong advice | Blaming vets | Veterinary treatments aren’t helping |
| ASF has no cure  ASF has no cure  ASF has no vaccine | Hopelessness | There is no medicine or vaccine for ASF |  |
